# A nonrandomized comparison of off -pump versus on-pump coronary bypass surgery in Egyptian patients

**DOI:** 10.1186/cc9430

**Published:** 2011-03-11

**Authors:** H El-Abd, S Salah

**Affiliations:** 1 Cairo University Hospitals, Cairo, Egypt; 2 Police Authority Hospital, Cairo, Egypt

## Introduction

Coronary artery bypass grafting (CABG) has traditionally been performed with the use of cardiopulmonary bypass (ONCAB). CABG without cardiopulmonary bypass (OPCAB) might reduce the number of complications. Thus, this study aims to compare between on-pump and off-pump surgery concerning postoperative morbidity and mortality, also to evaluate 6-month graft patency in Egyptian patients.

## Methods

This is a nonrandomized single-centre control trial prospectively conducted on 65 patients who were subjected to coronary artery bypass surgery followed by stay in the Open Heart Intensive Care Center of the Police Authority Hospital, in the period from July 2009 to January 2010. Patients were divided into two groups; group A: 25 patients underwent ONCAP, and group B: 40 patients underwent OPCAB. All of the demographic, operative and postoperative data were prospectively collected and analyzed statistically. Six months later, the patients underwent coronary angiography.

## Results

There was no significant difference between both groups intraoperatively concerning arrhythmias, blood transfusion, and hemodynamic support. Off-pump patients had a significantly higher mean number of constructed grafts than in the ONCAB group (mean, 3.30 ± 0.88 vs. 2.84 ± 0.80, *P *= 0.02). There were no significant differences between off-pump and on-pump regarding postoperative blood loss, blood transfusion, length of the ICU and the hospital stay, the ventilation time, the use of IABP, renal complications, respiratory complications, and reopening. However, graft occlusion, MI, ventricular tachycardia, cardiogenic shock, and disturbed conscious level significantly occurred in the OPCAB group. Postoperative mortality rate was significantly higher in the OPCAB group than in the ONCAB group (15% vs. 0%, *P *= 0.046). Follow-up angiograms in 40 patients (61.5%) who underwent 124 grafts revealed no significant difference between off-pump and on-pump groups regarding overall rate of graft patency (83.5% vs. 84.4%, *P *= 0.84). No mortality was reported in both groups at 6-month follow-up.

## Conclusions

There was a higher incidence in postoperative complications and mortality in off-pump procedure than the on-pump. At 6-month follow-up, no significant differences between both techniques were found in graft patency and mortality.
